# Innovations in the Management of Vaginal Cancer

**DOI:** 10.3390/curroncol29050250

**Published:** 2022-04-27

**Authors:** Anjali Kulkarni, Nupur Dogra, Tiffany Zigras

**Affiliations:** 1 Division of Gynecologic Oncology, University of Ottawa, Ottawa, ON K1H 8L6, Canada; akulkarni@toh.ca; 2 Department of Obstetrics & Gynecology, University of Toronto, Toronto, ON M5G 1E2, Canada; n.dogra@mail.utoronto.ca; 3 Trillium Health Partners, Division of Gynecologic Oncology, Mississauga, ON L5M 2N1, Canada

**Keywords:** vaginal carcinoma, vaginal tumors, vaginal cancers, human papilloma virus, surgery, external beam radiation, brachytherapy, concurrent chemoradiation, immunotherapy

## Abstract

Primary vaginal cancer is a rare gynecologic malignancy. Given the rarity of the disease, standardized approaches to management are limited, and a great variety of therapeutic conditions are endorsed. This paper reviews advances in surgical approaches, radiation, chemoradiation, and immunotherapy. Advances in surgical management including the increasing use of laparoscopic and endoscopic approaches, as well as the novel techniques in vaginal reconstruction, are reviewed. Concurrent chemoradiation remains a mainstay of treatment for vaginal cancer and has improved local control of disease and overall survival. Additionally, with metastatic disease or situations where toxicity from CCRT is unacceptable, systemic therapies including immunotherapy approaches are reviewed.

## 1. Introduction

Primary vaginal cancer is rare and represents 1 to 2% of all gynaecological malignancies [1]. In Canada, 1020 women are diagnosed, and 368 deaths occur annually from vaginal cancers [2]. According to the International Federation of Gynecology and Obstetrics (FIGO), vaginal cancer is strictly defined as cancer found in the vagina without clinical or histologic evidence of cervical or vulvar cancer, or a prior history of these cancers within 5 years [1]. Primary lesions are classified as vaginal cancer only after exclusion of other origins. By convention, tumors in the vagina that extend to, or involve the cervix, are classified as cervical cancer [1]. 

Given the rarity of vaginal cancer, there are no randomized control trials to guide treatment decisions, and clinical care guidelines are based on limited retrospective and comparative studies [3,4]. As such, there is no standardized approach to the management and treatment of vaginal cancer, and a great variety of therapeutic strategies are endorsed for this condition. 

The objective of this paper is to review the published literature on advances in treatment for vaginal cancer with a focus on innovations in surgical approaches, as well as advances in radiation treatment (RT), chemoradiation (CCRT), and systemic therapies. 

### 1.1. Epidemiology and Risk Factors

Vaginal cancer rates increase with age and peak incidence occurs in the sixth and seventh decades of life [3]. Less than 15% of patients are diagnosed below the age of 50 [3], and the average age at the time of diagnosis is 67 [5]. When vaginal malignancy is found in younger women, it is usually linked to cervical cancer [1].

Cancer found in the vagina is often suggestive of metastatic disease as 80 percent of vaginal neoplasms are secondary tumors from other primary malignancies of the endometrium, cervix, or vulva [1]. Vaginal metastasis can also occur with non-gynaecological malignancies such as kidney, breast, or lung; although, this is extremely rare [6,7,8].

Of histologic forms, the predominant subtype is squamous cell carcinoma (SCC), which comprises 90 percent of all primary vaginal cancer cases [1]. Adenocarcinomas account for 8 to 10 percent of cases and have a peak incidence between 17 and 21 years of age [3]. Clear cell adenocarcinoma of the vagina is rare and occurs most commonly in patients below 30 years of age; it is associated with adenosis and in utero exposure to diethylstilbestrol (DES) [3]. Melanomas, sarcomas, and lymphomas of the vagina are extremely rare [1]. 

As with cervical cancer, vaginal cancer has a strong association with the human papillomavirus (HPV) as nearly two-thirds of cases are related to HPV16 [9]. Co-factors include immunosuppression and smoking [1]. Other risk factors for SCC of the vagina are: five or more sexual partners, first intercourse before 17 years of age, low socioeconomic status, a history of genital warts, prior abnormal cytology, and prior hysterectomy [3,9]. Patients with a history of cervical cancer have an increased risk of developing vaginal cancer as these sites share exposure and susceptibility to HPV-related infections [3]. 

Previously, a three-tiered classification system was used for precancerous HPV-associated vaginal lesions, i.e., vaginal intraepithelial lesion (VAIN) 1 to 3 [1]. In 2014, the World Health Organization (WHO) replaced VAIN with squamous intraepithelial lesion (SIL) [1]. These lesions were divided into two categories: low grade (LSIL) and high grade (HSIL). LSIL (previously VAIN 1) is associated with either low- or high-risk HPV and represents transient infections that may regress. In contrast, HSIL represents transforming high-risk infections (previously VAIN 2–3). The risk of progression from HSIL to invasive cancer ranges from 2 to 12% [1].

### 1.2. Anatomy

**Figure 1:**
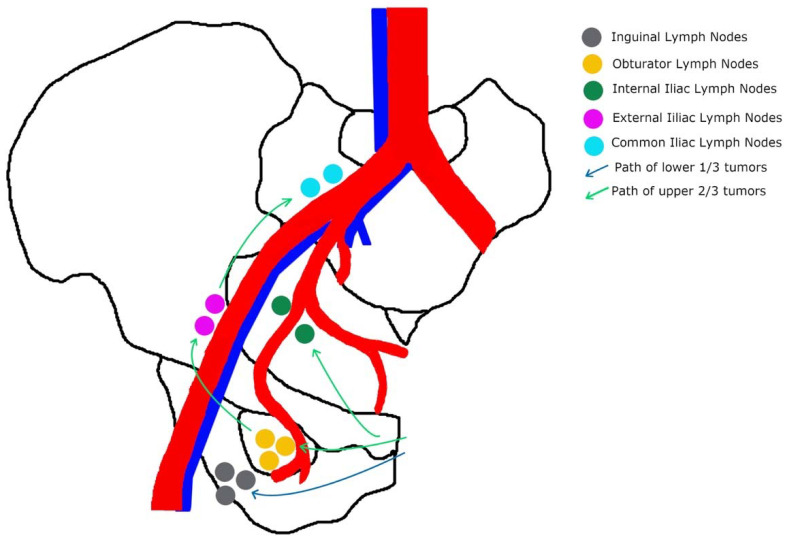
Specific pathway of the lymphatic drainage of vaginal tumors: the upper two-thirds of the vagina drain primarily to pelvic nodes; and the lower one-third drains into the inguinal nodes.

The most common site for vaginal cancer is the vaginal apex or upper third of the vagina, which accounts for 56 percent of cases [1,10]. Subsequently, the lower third of the vagina accounts for 31 percent of cases, and lastly, the middle third accounts for 13 percent [10]. Figure 1 outlines the lymphatic drainage of vaginal cancer. Tumors localized in the upper two-thirds of the vagina most likely drain into the pelvic lymph nodes, including the obturator, internal iliac, and external iliac; while tumors in the lower third drain into the inguinal lymph nodes [9,10]. Lesions in the mid-vagina may follow both the pelvic and groin routes [9]. In addition to lymphatic spread, vaginal cancer also spreads directly to para-cervical tissues, to the vulva, and by contiguity to the bladder and rectum [11]. Hematogenous spread to the lung, liver, and bone is also seen in late manifestations [1]. Thus, the route of metastasis varies with site and extension of the primary tumor.

### 1.3. Prognostic Factors

The stage of disease at the time of diagnosis is the most important prognostic factor [3,4,9]. The 5-year survival rate for FIGO stage I and II is favorable as compared to stage III and IV, which confers a poor prognosis [4,9]. The stage at diagnosis was found to be the only factor for predicting recurrence [3,4]. 

Beyond staging, the other factors that negatively affect prognosis include tumor size >4 cm, older age, and tumor location outside of the upper third of the vagina [9,11]. Hiniker et al. showed tumor size >4 cm is associated with decreased local control of disease and worsened overall survival [4]. Histological subtype also affects prognosis as adenocarcinomas have a worse prognosis than SCC [9]. 

Conversely, better clinical outcomes are seen with high-risk HPV DNA and low Ki-67/MIB-1 expression, which are favourable prognostic values. Ki-67 is a protein found in the nuclei of growing cells and is associated with cellular proliferation. MIB-1 is a monoclonal antibody for Ki-67 antigen and, thus, a marker of mitotic rate, and it has been important in many gynecological cancers, including ovarian and vaginal cancers [9].

### 1.4. Diagnosis and Staging

There are no routine screening programs for vaginal cancer. However, Pap smears are effective in detecting asymptomatic lesions. In cases of abnormal results without gross cervical lesions, a colposcopy of the vagina should be performed.

Painless vaginal bleeding and vaginal discharge, as well as post-coital or postmenopausal bleeding, are common signs of vaginal cancer [3,11]. There may be involvement or compression of nearby organs resulting in urinary complaints such as retention, dysuria, hematuria, or gastrointestinal symptoms such as tenesmus, constipation, or melena. Locally advanced disease is also associated with pelvic pain. 

Approximately 5 to 10% of women remain asymptomatic and disease is detected during routine physical examination or following an abnormal Pap test [3]. A biopsy will confirm a diagnosis. A thorough physical examination is an essential component for the evaluation of the local extent of the disease. Specifically, noting the location, gross morphology, sites of involvement, and dimensions of the visible and palpable tumor will be important for the management of the disease [3]. Visualization of the entire vagina can be performed under general anesthesia if needed. 

Staging is performed clinically rather than surgically; it is based on the FIGO staging system. Stage I disease is limited to the vaginal wall. Stage II disease involves subvaginal tissue, but it does not extend to the pelvic wall. Stage III tumor extends to the pelvic wall, and Stage IV tumor extends beyond the true pelvis or involves the mucosa of the bladder or rectum.

With imaging not incorporated into the FIGO staging system, the extent of disease in each stage can be variable. Due to this, FIGO recommends that where available, imaging findings guide management [1]. As reviewed by Jhingran, MRI is recommended for assessment of tumor volume, extension of disease, and recurrence and complications [9]. As extrapolated from cervical cancer data, MRI is more sensitive in detecting tumor size, and paravaginal or parametrial involvement [1]. PET/CT is also superior to other modalities for assessing lymph nodes and detecting recurrence [1,9]. 

## 2. Materials and Methods

A literature search was performed in the Ovid MEDLINE database for articles on the treatment and management of vaginal cancer. The following keywords were used in various search algorithms: “vaginal neoplasm”, “vaginal cancer”, “vaginal tumor”, “vaginal malignancy”, “vaginal carcinoma”, “radiation therapy,” “chemoradiation”, “brachytherapy”, “immunotherapy”, and “innovations”. Original research, case reports, abstracts, and review papers published on the topic from 2010 to 2022 were considered. Further references found within the articles that were relevant to the subject were also used. Articles reviewed were published in English. 

## 3. Advances in Surgical Management

In the treatment of vaginal cancer, surgery has a limited role given the proximity of the vagina to vital organs such as the bladder, urethra, and rectum. As such, surgery is considered in selected cases of: (1) small stage I and II tumors that are confined to the upper posterior vagina; (2) stage IV disease with recto-vaginal or vesico-vaginal fistulas; and (3) central recurrence after RT [1,11]. 

Surgical resections for vaginal cancer include local tumor excision (LTE), vaginectomy (partial, total, and radical), and pelvic exenteration. The type of surgery depends on the location and extent of the initial tumor. Zhou et al. recently compared the effectiveness of LTE versus vaginectomy for stage I and stage II vaginal carcinoma using SEER data from 2004 to 2016. Although LTE is more commonly performed over vaginectomy as a less aggressive procedure and to preserve sexual function, vaginectomy resulted in significantly prolonged survival and should be the preferred treatment regardless of radiotherapy status [12].

FIGO recommends that in patients with stage I disease involving the upper posterior vagina and with the uterus in situ, a radical hysterectomy and upper vaginectomy are performed with an aim for 1 cm negative margins, and a pelvic lymphadenectomy to assess for nodal disease [1]. If a hysterectomy has been previously performed, a radical upper vaginectomy and pelvic lymphadenectomy are more appropriate [1]. There are currently no data on the use of sentinel lymph node (SLN) mapping in the assessment of patients with vaginal cancers [13]. However, a small study of pre-treatment lymphatic and SNL mapping with lymphoscintigraphy in patients with vaginal cancer demonstrated the feasibility of this approach wherein one SLN was identified in 79% of patients [13,14].

Yin et al. previously described five cases of vaginal carcinoma in the upper third of the vagina, which were treated with radical hysterectomy, vaginectomy, pelvic lymphadenectomy, and sigmoid vaginoplasty. All patients recovered well after surgery with no recurrence or delayed complications during a mean follow-up of 22.8 months, and all patients were satisfied with their sexuality [11,15]. 

In patients with stage I disease involving the lower vagina, a radical wide local excision is performed with 1 cm margins, and with bilateral groin node dissection [1]. Novel techniques for local excision have also been reported, such as the use of natural orifice transluminal endoscopic surgery (NOTES) for the management of vaginal carcinoma. In their case report, Smink et al. discuss the clinical application of transvaginal endoscopic microsurgery in a woman with residual disease after treatment with CCRT for SCC of the vagina [16]. Despite the difficulty of operating in tissue with post-radiation effect, the tumor was excised with clear surgical margins without damage to the rectum. The patient was discharged from the hospital two days after the procedure and recovered without complication.

Further, the integration of laparoscopic procedures has facilitated enhanced visualization of the operative space and distinct pelvic anatomical structures. Jiang et al. utilized laparoscopic radical parametrectomy (LRP) in comparison to abdominal radical parametrectomy (ARP) as an alternative to radiotherapy in treating invasive cervical cancer, vaginal apex cancer, and endometrial cancer. LRP was superior to ARP in terms of shorter operative time, less blood loss, and shorter hospital stay [17].

In young patients who are planning to undergo primary RT, a pre-treatment laparotomy or laparoscopy is undertaken for ovarian transposition to preserve ovarian function [11]. In selected cases, surgical staging and resection of any bulky positive lymph nodes may be performed as part of staging and treatment planning [11]. Alternatively, Mabuchi et al. presented a case report in which a 36-year-old non-parous woman with a solid tumor (confirmed as vaginal SCC on biopsy) wished to retain her fertility and, thus, was treated with neoadjuvant chemotherapy and then partial vaginectomy [18]. She went on to receive the same chemotherapy for adjuvant therapy and remained disease-free at 14 months.

Primary pelvic exenteration should also be considered for select patients. In patients with stage IV disease, particularly if a rectovaginal or vesicovaginal fistula is present, an exenteration can be performed for palliation [1]. Similarly, pelvic exenteration is also usually necessary in patients with central recurrence after RT [1]. Pelvic exenteration may be combined with pelvic lymphadenectomy or neoadjuvant radiation. Bilateral inguino-femoral groin dissection should be considered if the lower third of the vagina is involved [1]. In rare cases, a patient who received pelvic RT for another malignancy, such as endometrial cancer, may have a primary vaginal cancer arise in the radiated field. If there is no evidence of metastatic disease, pelvic exenteration may be considered. 

Given the radical surgical options for primary vaginal carcinoma, which involve partial or complete resection of the vagina, patients can experience sexual dysfunction after surgery. This must be discussed with patients and if preservation of sexual function is desired, the patient will require vaginoplasty. In the past decade, this has been a growing area of interdisciplinary literature from gynaecology to plastic surgery, as multiple vaginal reconstructive techniques have been reported [19,20,21,22,23,24,25]. Vaginal reconstructive techniques include skin grafts, peritoneal grafts, abdominal and gracilis myocutaneous flaps, and, more recently, bowel flaps. The choice of technique is important for obtaining function and aesthetics. However, complications for these procedures remain high, including a graft failure rate of 13–38% for myocutaneous flaps [26,27]. Other complications also include flap necrosis, donor site scarring, wound dehiscence, abscess formation, prolapse, sepsis, and death [27,28]. 

Yao et al. performed a pilot study to assess the feasibility of using the pelvic peritoneum to create a neovagina and compared this to the use of the sigmoid colon [25]. They found that laparoscopic vaginoplasty using the peritoneum was simpler and more feasible as compared to using the sigmoid colon for management of stage I primary vaginal carcinoma. The benefits included shorter operative time, no bowel disturbance, production of a hygienic vaginal environment, and satisfactory sexual function and oncologic outcomes comparable to sigmoid colon vaginoplasty [25]. However, these patients typically had more shrinkage of the peritoneum, resulting in a shorter length of the neo-vagina and required use of a vaginal mold for 6 months after surgery leading to delays in resuming sexual activity [25]. 

## 4. Advances in Radiation Therapy

Definitive RT based on external beam (EBRT) and/or brachytherapy (BT) is considered a standard approach to treatment for vaginal cancer, especially for locally advanced cases. There are no prospective trials on the role of RT for primary vaginal cancer [1,9,29,30].

Guerri et al. previously published a systematic review on the role of definitive RT in vaginal cancer [29]. A total of 13 studies were included in this review, all with a variety of RT techniques for the treatment of squamous cell carcinoma and adenocarcinoma of the vagina. The 5-year overall survival reported in these studies differed based on stage as follows: I: 65.0–92.0%; II: 25.0–82.0%; III: 26.0–68.0%; and IV: 0.0–50.0% [29]. Characteristics associated with better outcomes included early stage of disease, small (<4 cm) tumor size, previous hysterectomy, and high pre-treatment hemoglobin levels [29]. 

Yang et al. performed a retrospective review of 124 patients at their institution treated for primary vaginal cancer [30]. Of these, a total of 86 patients underwent primary EBRT with a dose ranging from 45 to 66 Gy. For patients who are stage I–II, overall survival outcomes with primary surgery are comparable to primary RT [30].

Another review included 68 patients with vaginal cancer who were treated with either radical or adjuvant RT over the course of 20 years at one institution. The majority of patients were treated with EBRT or EBRT in combination with BT. As presented by Guerri et al., the stage was the most common prognostic factor predicting disease-specific survival (DSS) [29]. The type of treatment patients received was not shown to be clinically significant [31]. Similarly, a large retrospective review by Frank et al. demonstrated excellent outcomes with definitive RT in the form of either EBRT alone or in combination with BT. This highlights the importance of RT to be individualized based on patient factors, without compromising outcomes [9,31,32].

Similar to cervical cancer, image-guided RT using either CT or MRI is being used more frequently for the treatment of vaginal cancer. When compared to two-dimensional BT, three-dimensional imaging has led to a 10% improvement in survival in cervical cancer [33]. Depending on the depth of invasion of the primary lesion, image-guided interstitial BT has also been utilized [34]. While the sample size is smaller and the follow-up time is shorter in vaginal cancer studies, a similar improvement is seen with the use of more advanced imaging techniques. As well, a decrease in toxicities is also demonstrated [33]. 

## 5. Advances in Chemoradiation Therapy

Due to the similarities in the histology, HPV association, and natural history of cervical cancer and vaginal cancer, treatment decisions for vaginal cancer often extrapolate from evidence for cervical cancer. CCRT has been increasing in use for the treatment of vaginal cancer, mirroring the rates for the treatment of cervical cancer [35,36].

Various retrospective studies have demonstrated a potential benefit of the use of CCRT for vaginal cancer. The most common agents used are cisplatin and 5-fluorouracil. While the impact on OS varies among the studies, they overall demonstrate that CCRT is well-tolerated by patients, with minimal grade 3–4 toxicities seen [36,37,38,39].

A retrospective cohort study conducted from 1998 to 2011 based on data from the National Cancer Data Base looked at 8222 patients with vaginal cancer treated with radiation. This was the largest population-based retrospective analysis that has been completed, investigating the impact of CCRT on overall survival for vaginal cancer. Of the total sample, 3932 patients received CCRT and 4154 received RT alone. This study demonstrated that since 1998, the rate of use of CCRT has tripled to nearly 60% in 2011. As well, CCRT was demonstrated to improve 5-year OS by 6.9%, which is comparable to the OS benefit of CCRT for cervical cancer. CCRT was shown to be independently associated with an improvement in OS for all stages of the disease [35].

## 6. Advances in Systemic Treatments

RT and CCRT is the standard treatment for early-stage vaginal cancer; side effects and toxicities tend to be well-accepted and tolerated by the patients. However, in rare circumstances, vaginal cancer can present in younger patients who wish to preserve fertility and sexual function, leading to the need for individualized treatment options. In these cases, systemic therapy may initially be considered [40,41]. In addition, while most patients with vaginal cancer present with early-stage or locally advanced disease, approximately 13% present with stage IV disease, necessitating systemic therapy. As well, distant recurrences rates range from 7 to 33%, usually occurring late in the natural history of the disease and may be considered for treatment with systemic therapy [42].

In these situations, where the disease is not responsive or amenable to radiation or surgery, vaginal cancers remain challenging to treat. There is currently no consensus on effective regimens for the systemic treatment of vaginal cancer [42,43,44].

### 6.1. Chemotherapy

Due to the rarity of vaginal cancer, and even more so, the rarity of advanced vaginal cancer, prospective data and clinical trial data on the efficacy of systemic treatments are sparse. The literature on the use of chemotherapy for vaginal cancer is mainly in the form of case reports and anecdotal data. The current evidence is summarized in Table 1.

One phase II trial on the use of cisplatin in recurrent or metastatic vaginal cancer included 22 patients with various histologies [42]. An overall response rate of 6.25% was demonstrated in the 16 patients with squamous histology. 

Otherwise, several case reports exist in the literature addressing the use of various chemotherapy regimens in the neoadjuvant setting, as well as in the setting of fertility-sparing definitive treatment [18,42,45,46]. Mabuchi et al. and Umesaki et al. report on patients with early-stage vaginal cancers treated with primary platinum-based chemotherapy due to their desire to preserve fertility. In both cases, a complete response was demonstrated with chemotherapy alone [18,45]. Similarly, Lv et al., Diao et al., and Panici et al. report on patients with stage I–II vaginal cancer who wished to avoid RT to maintain sexual function. They were treated with neoadjuvant chemotherapy, followed by radical surgery, with good effect [40,41,46]. 

### 6.2. Immunotherapy

With variable response rates seen with standard chemotherapies, there is a need to explore other systemic treatment options. The role of immunotherapy is of interest, particularly for HPV-related cancers such as vaginal cancer. Due to the rarity of the disease, patients with vaginal cancer are often grouped together with vulvar cancer in clinical trials.

One phase II basket trial on the use of pembrolizumab, a PD-1 inhibitor, in the treatment of recurrent or metastatic disease included two patients with vaginal cancer. Both patients demonstrated recurrent disease with distant metastases and had been previously treated with radiation and multiple lines of chemotherapy. One patient demonstrated stable disease in response, while the second had progression on treatment. Treatment with pembrolizumab was well-tolerated, with grade 2 fatigue as the only treatment-related adverse event reported [43].

The CheckMate 358 trial was a phase I/II trial assessing nivolumab in patients with recurrent or metastatic tumors of the cervix, vagina, and vulva [44]. Specifically, patients who were HPV positive, or HPV unknown, were included, while patients who were HPV negative were excluded. Five patients with recurrent or metastatic vulvar or vaginal cancer were included in the study; they were grouped together for the final analysis. Of these, one patient demonstrated a partial response and two demonstrated stable disease. One was diagnosed with disease progression while on treatment. The 12- and 18-month overall survival rates were 40.0% and 20.0%, respectively; the 6-month progression-free survival rate was 40.0% [44].

Prognosis is poor in cases of stage IV or recurrent metastatic disease. Treatments with standard chemotherapy have a low response rate. With more evidence for the efficacy of neoadjuvant chemotherapy and immunotherapy in cervical cancer, these therapies are being utilized more frequently in vaginal cancer. Overall, further investigation is required on the efficacy of systemic treatments in the form of chemotherapy and immunotherapy in the setting of vaginal cancer.

## 7. Special Circumstances

The majority of treatments addressed so far pertain to squamous cell carcinoma and adenocarcinoma of the vagina, which are the most common histologies. Significant advances have also been made in the treatment of vaginal melanoma and vaginal botryoid rhabdomyosarcoma (RMS).

### 7.1. Vaginal Melanoma

Less than 4% of all vaginal cancers are primary melanomas [47]. The majority of vaginal melanomas are in the distal third of the vagina and on the anterior wall. Due to their hidden location, in addition to their vascular connection to a lymphatic network, vaginal melanomas are more likely to present at an advanced stage and have a higher mortality rate [47]. Given the rarity of this disease, there are no randomized trials to guide treatment decisions. Retrospective reviews on the management of vaginal melanomas demonstrate they are treated with a combination of surgery, radiation, and systemic treatment. Similar to more common histologies of vaginal cancer, surgery has a role in the early stages. The radicality of the surgery required is debated in the literature [48,49]. In addition, while radiation is the mainstay of treatment for squamous cell vaginal cancer, its impact on vaginal melanoma has demonstrated mixed results [50].

Traditional systemic treatments have had limited efficacy in treating metastatic or recurrent mucosal melanomas (including vaginal). While immunotherapy has demonstrated promise in the treatment of cutaneous melanomas, its efficacy in mucosal melanomas needs further exploration [50,51]. A large, pooled analysis of six clinical studies demonstrates the safety of ipilimumab, in combination with nivolumab, in treating mucosal melanomas. However, the proportion of these patients with vaginal melanomas is not reported [51]. Given the rarity of vaginal melanomas, it is recommended that all cases be reviewed in a multidisciplinary case conference with the input of experts in the management of melanoma.

### 7.2. Vaginal Botryoid Rhabdomyosarcoma

In the pediatric population, rhabdomyosarcomas (RMS) arising from the genital tract account for approximately 3.5% of RMS [52]. They are the most common vaginal malignancy in childhood [53]. Embryonic RMS are subdivided into: classic, botryoid, and spindle cell. Of these, vaginal botryoid RMS is most commonly present in infancy or childhood [53]. Symptoms of vaginal botryoid RMS include vaginal bleeding, as well as a mass protruding from the vagina with a grape-like appearance [52,53]. The management and prognosis of vaginal botryoid RMS have changed significantly over time. Previously, the traditional treatment included radical pelvic surgery (total vaginectomy or exenteration) and radiation, which has resulted in poor prognosis, as well as significant morbidity. Unlike more typical vaginal carcinomas, chemotherapy has high efficacy against botryoid RMS. Because of this, multimodal treatment involving chemotherapy and more conservative surgery is becoming the mainstay of treatment [53]. The overall survival rates for local disease are now 97%, with preservation of sexual function and fertility [52,53]. Referral to specialized pediatric centers, where possible, should be considered to manage this rare condition.

## 8. Conclusions

Treatment of vaginal cancers remains a challenge as high-quality evidence from prospective randomized trials remains elusive due to the rarity of the disease. Techniques for CCRT, the mainstay of treatment for vaginal cancer, have been advancing, resulting in improvements in survival, as well as decreasing toxicities. In the rare circumstance where CCRT is not possible, either due to metastatic disease or unacceptable toxicity, surgery and systemic therapies are utilized. This review covers various surgical techniques, as well as systemic therapies that have shown promise in the treatment of select cases of vaginal cancer. Further research will help to guide individualized treatment decisions in the future. Given the rarity of vaginal cancers, collaborative, multicenter trials are needed to study treatment effects, and national registries should be used to study the outcomes. 

## Figures and Tables

**Table 1:** Case series and case reports for systemic chemotherapy for treatment of vaginal cancer.

Series/Report	Chemotherapy Agents	Stage	Notes
Diao (2017)	Irinotecan Cisplatin	Stage I, *n* = 2 Stage II, *n* = 1	All 3 patients had complete response after 2–4 cycles 2 patients underwent surgery after with no residual disease on pathology All patients disease-free at 45, 48, and 6 months, respectively
Mabuchi (2015)	Irinotecan Nedaplatin	Stage I, *n* = 1	No tumor identified after 4 cycles Partial vaginectomy after demonstrated only VAIN3 Disease-free at 14 months
Lv (2010)	Bleomycin Cisplatin	Stage II, *n* = 1	Partial response to 2 cycles of neoadjuvant chemotherapy Radical surgery and vaginal reconstruction after 2 cycles 4 further cycles of adjuvant chemotherapy Disease-free at 30 months
Benedetti (2008)	Paclitaxel Cisplatin	Stage II, *n* = 11	All patients received 3 cycles of chemotherapy, followed by surgery 3 patients had complete clinical and pathological response to chemotherapy, 7 had partial response, 1 had stable disease 8 patients disease-free at median follow-up of 75 months
Umesaki (1999)	Irinotecan Cisplatin	Stage II, *n* = 1	No tumor identified on MRI after one cycle of neoadjuvant chemotherapy Radical surgery performed after 1 cycle, pathology negative
Thigpen (1986)	Cisplatin	Stage IV or recurrent, *n* = 16	16 patients with recurrent or metastatic squamous cell carcinoma of the vagina 1 complete responder (ORR = 6.25%)

## Data Availability

Not applicable.
